# Multiple Consequences Induced by Epidermally-Located Anthocyanins in Young, Mature and Senescent Leaves of *Prunus*

**DOI:** 10.3389/fpls.2018.00917

**Published:** 2018-07-02

**Authors:** Ermes Lo Piccolo, Marco Landi, Elisa Pellegrini, Giovanni Agati, Cristiana Giordano, Tommaso Giordani, Giacomo Lorenzini, Fernando Malorgio, Rossano Massai, Cristina Nali, Giovanni Rallo, Damiano Remorini, Paolo Vernieri, Lucia Guidi

**Affiliations:** ^1^Department of Agriculture, Food and Environment, University of Pisa, Pisa, Italy; ^2^“Nello Carrara” Institute of Applied Physics, CNR, Sesto Fiorentino, Italy; ^3^Trees and Timber Institute, CNR, Sesto Fiorentino, Italy

**Keywords:** anthocyanin, leaf ontogenesis, photoprotection, pNPQ, photo-oxidative stress, red-leafed *Prunus*, senescence, xanthophyll

## Abstract

Anthocyanic morphs are generally less efficient in terms of carbon gain, but, in turn, are more photoprotected than anthocyanin-less ones. To date, mature leaves of different morphs or leaves at different developmental stages within the same species have generally been compared, whereas there is a lack of knowledge regarding different stages of development of red *vs*. green leaves. Leaves (1-, 7-, and 13-week-old) of red- (RLP) and green-leafed (GLP) *Prunus* in terms of photosynthetic rate, carbon metabolism and photoprotective mechanisms were compared to test whether anthocyanin-equipped leaves perform better than anthocyanin-less leaves and whether photoprotection is the primary role of epidermally-located anthocyanins, using for the first time a recently-developed parameter of chlorophyll fluorescence (qPd). GLP leaves had a higher photosynthetic rate in 1- and 7-week-old leaves, but RLP leaves performed better at an early stage of senescence and had a longer leaf lifespan. Anthocyanins contributed to leaf photoprotection throughout the leaf development, but were tightly coordinated with carotenoids. Besides photoprotecting, we propose that epidermal anthocyanins may be principally synthetized to maintain an efficient carbon-sink strength in young and senescent leaves, thus extending the RLP leaf lifespan.

## Introduction

Although the ecological role of foliar anthocyanins (Gould et al., 2009; Hughes and Lev-Yadun, 2015; Landi et al., 2015; Menzies et al., 2016) has been thoroughly investigated, their functional significance is still an open issue (Gould, 2004; Hughes et al., 2005; Manetas, 2006; Menzies et al., 2016). The cost/benefit ratio that the plant maintains for the investment of carbon skeletons is still unknown. These carbon skeletons are used for the biosynthesis of anthocyanins, which are reclaimed from the primary metabolism usually addressed to plant growth (Hughes et al., 2005). Leaf anthocyanins can be accumulated in the lower and upper epidermis (Hughes and Smith, 2007; Merzlyak et al., 2008; Landi et al., 2013, 2014), the palisade and spongy mesophyll, or parenchymal cells (Hughes et al., 2007; Kyparissis et al., 2007). Their biosynthesis (Cominelli et al., 2008; Loreti et al., 2008; Albert et al., 2015) and degradation (Zipor et al., 2015) are tightly regulated and their localization may change during leaf ontogenesis (Merzlyak et al., 2008). Depending on the ontogenetic stage of the leaf, the reddish coloration can be a permanent or transitory trait, especially during the juvenile and senescent phases (Kytridis et al., 2008; Zeliou et al., 2009).

Anthocyanin biosynthesis under abiotic stress such as low temperatures, salinity, nutrient deficiency (Chalker-Scott, 1999; Archetti et al., 2009; Landi et al., 2015) has always been a feature of land plants (Albert et al., 2018). The most accepted hypothesis is that they protect the leaf from excessive light radiation, screening the photosynthetic apparatus from supernumerary (in row) green > yellow > blue photons (500–600 nm) (Gould et al., 2018). Irrespectively of their chemical structure, all red anthocyanins absorb green light (Harborne, 1958) and therefore fewer green photons reach chloroplasts in red than in green leaves (Landi et al., 2014; Nichelmann and Bilger, 2017).

The capacity to attenuate green light depends on the histological location of the anthocyanins (Neill and Gould, 1999; Hughes et al., 2014), and epidermally-located anthocyanins seem better located to photoprotect the subjacent mesophyll from photoinhibition and/or to ensure the rapid recovery of photosystem II (PSII) after a photoinhibitory condition (Hatier et al., 2013; Logan et al., 2015; Buapet et al., 2017; Tattini et al., 2017). However, several papers have failed to find a protective function of these pigments (e.g., Burger and Edwards, 1996; Kytridis et al., 2008; Zeliou et al., 2009; Liakopoulos and Spanorigas, 2012).

Besides the “mere” presence of anthocyanins, red leaves commonly exhibit typical morpho-anatomical traits (less compact mesophyll and leaf thickness; Kyparissis et al., 2007; Tattini et al., 2014), as well as physiological (lower stomatal conductance; Landi et al., 2013) and biochemical features (higher chlorophyll content and lower chlorophyll a:b ratio on a weight basis; Lichtenthaler et al., 1981; lower xanthophyll content and de-epoxidation state; Hughes et al., 2012; Landi et al., 2015; Logan et al., 2015), which usually overlap with those found in shade plants, i.e., the “shade acclimation syndrome” *sensu* Lambers et al. (1998) and Manetas et al. (2003). Red leaves thus often have a lower photosynthetic rate than their green counterparts, perhaps due to the competition in light harvesting between anthocyanins and chlorophylls (Gould et al., 2002; Hatier et al., 2013). However, this lower photosynthetic ability has almost been exclusively demonstrated in mature leaves, whilst no reports have evaluated the photosynthetic performance of red vs. green leaves throughout ontogenesis.

The massive accumulation of anthocyanins may also offer an alternative way of reducing the accumulation of hexoses, thus delaying the sugar-induced early senescence of leaves, as observed in red vs. green autumn leaves (Feild et al., 2001; Hoch et al., 2003; Schaberg et al., 2003). Hexose accumulation is known to repress or down-regulate the expression of photosynthetic genes (Paul and Pellny, 2003; Granot et al., 2014; Lastdrager et al., 2014), and glucose (Moore et al., 2003) and fructose (Pourtau et al., 2006) accumulation can induce an anticipated senescence in leaf. However, the intimate relationship between carbohydrate content and anthocyanin production still needs clarifying.

As young and senescent leaves are usually more vulnerable to photoinhibition (Juvany et al., 2013), epidermally-located anthocyanins, photoprotecting the subjacent mesophyll cells (only partially functioning), may perhaps improve the photosynthetic performance of red morphs, making them more competitive in limiting conditions. To test this hypothesis, we determined leaf gas exchange, chlorophyll *a* fluorescence and pigment content in two morphs of *Prunus cerasifera* with permanent red (var. Pissardii) or green (clone 29C) leaves from juvenility (1 week-old leaves) to (early) senescence (13 week-old leaves).

We are aware that beside the obvious presence of foliar anthocyanins, other physiological and biochemical features might have differed between the two morphs. However, the different genetic background of the two morphs may only have been of secondary importance to our core results, which mainly depended on the different leaf pigmentation and the adaptive traits connected to the constitutive presence of foliar anthocyanins. In addition, it is almost impossible for tree species to operate a knockout approach to obtain targeted anthocyanin-less mutants as those obtained in Arabidopsis, which were used for the first time to unequivocally test the photoprotective role of these pigments (Gould et al., 2018).

## Materials and methods

### Plant material

A total of 200 three-year-old *P. cerasifera* saplings (clone 29C, GLP; var. *Pissardii*, RLP) were purchased from an Italian nursery (Vivai Battistini, Cesena, IT). Stems of both morphs were grafted onto 29C rootstock in November 2016. One month after grafting, red and green individuals were transplanted to 6.5-L pots in a growing medium containing a mixture of Einhetserde Topfsubstrat ED 63 standard soil (peat and clay, 34% organic C, 0.2% organic N and pH 5.8–6.0) and sand (3.5:1 in volume). They were maintained under greenhouse conditions until March 2017, when they were transferred to open field conditions at the Department of Agriculture, Food and Environment, University of Pisa, Italy (43°42′N 10°25′E).

During the experiments, plants were kept well-watered and fertilized. One week after the emergence (early July 2017, when maximum irradiance is experienced by the leaf), homogeneous leaves from 100 plants of both morphs, were marked to be followed throughout ontogenesis. Mean monthly temperatures and precipitations for the period are reported in Figure S1. At each sampling date (1, 7, and 13 weeks after leaf emergence), samples for biochemical analysis were collected at midday, immediately frozen in liquid nitrogen, and stored at −80 °C until analysis.

### Leaf gas exchange and chlorophyll *a* fluorescence analysis

Gas exchange measurements were determined from 10.00 to 14.00 h from five replicates (one leaf for each sapling) of emergent (1-week-old), mature (7-week-old) and early senescent (13-week-old) leaves, using a portable infrared gas analyser (Li-Cor 6400, Li-Cor Inc., Lincoln, NE, USA) following the procedure described by Guidi et al. (2017). Mitochondrial respiration in the light (${\text{R}}_{\text{d}}^{*}$) and the CO_2_ compensation point in absence of respiration, Γ^*^, were calculated using the Laisk method (von Caemmerer, 2000). Mesophyll conductance (g_m_) and chloroplastic CO_2_ concentration (C_c_) were estimated using the variable J method (Harley et al., 1992), through the combination of gas exchange and chlorophyll fluorescence analysis. The maximum carboxylation rate at substomata (V_cmax(Ci)_) and chloroplastic CO_2_ concentration (V_cmax(Cc)_), the maximum electron transport rate (J_max_) and the triose phosphate utilization (TPU) were measured by fitting Farquhar's equation (Farquhar et al., 1980).

Chlorophyll fluorescence was measured using a PAM-2000 fluorometer (Walz, Effeltrich, Germany) at the same time as the gas exchange measurements (five replicates), after 30 min of dark adaptation as reported in Degl'Innocenti et al. (2002). Ruban and Murchie method (2012) was used to estimate the parameter, qPd (photochemical quenching measuring in the dark), which allows to detect the earliest signs of photoinhibition (when qPd value is 1 corresponds to 100% of open RCIIs). qPd is calculated as:

$$\begin{array}{l} \text{qPd}=\left({\text{F}}_{\text{m}}\text{'}-F_{0}\text{'}\right)/\left({\text{F}}_{\text{m}}\text{'}-{\text{F}}_{0}{\text{'}}_{\text{calc}}\right) \end{array}$$

where F_m_' is the maximum level of fluorescence yield in the light and F_0_', and F_0_'_calc_ are the measured and calculated dark fluorescence levels, respectively. F_0_'_calc_ was determined according to Oxborough and Baker (1997):

$$\begin{array}{l} {\text{F}}_{0}{\text{'}}_{\text{calc}}=1/\left[1/{\text{F}}_{0}-1/{\text{F}}_{\text{m}}+1/{\text{F}}_{\text{m}}\text{'}\right] \end{array}$$

To determine the values of qPd, a Walz Junior-PAM fluorometer (Walz) with a monitoring leaf clip was used. A sequence of increasing actinic illumination steps (ranging from 0 to 1,500 μmol m^−2^ s^−1^) was used, each lasting 5 min (Ruban and Belgio, 2014). The routine was encoded as a batch programme that sets the saturation pulse for 600 ms and turns on the actinic light of the lowest intensity (90 μmol m^−2^ s^−1^) after 40 s of F_0_ determination in the presence of the low intensity far-red light. During illumination by actinic light (5 min), only two saturation pulses were applied at the second and fifth minutes of illumination needed to calculate NPQ and Φ_PSII_. After 5 min, the light was switched off immediately after applying the second saturation pulse. After 7 s of far-red light illumination, the saturating pulse was applied in the dark for 5 s, followed by the same cycle of actinic light illumination.

### Soluble sugar, sorbitol, starch, and nitrogen content

Sugar and polyol quantification were conducted according to slightly modified methods of Yusof et al. (2016) and Sotelo et al. (2014), respectively. For soluble sugar and polyol extraction, 100 mg of dried leaf samples were finely ground in a mortar, suspended in 10 mL of 80% aqueous ethanol (v/v), and placed in an ultrasonic water bath at 60°C for 30 min. The solution was centrifuged at 10,000 g for 10 min at 10°C, and the supernatant was filtered using a HPLC filter (pore size: 0.45 μm). Sucrose, glucose, fructose and sorbitol were quantified using K-SUFRG and K-SORB commercial kits (Megazyme, Wicklow, Ireland), following the manufacturer's protocol. The residual pellet of the centrifuged solution was used for starch quantification using commercial kit K-TSTA (Megazyme) according to the manufacturer's protocol.

Total N content was determined following the Kjeldahl method (Mitchell, 1998) and expressed as the percentage dry weight (DW). According to Sanz-Pérez et al. (2009), Nitrogen Resorption Efficiency was calculated as (N_m_ − *N*_s_)/N_m_ × 100, in which N_m_ is nitrogen content in the mature leaf and N_s_ is that contained in the early senescent leaf. For RLP the index was also calculated at 17 weeks, when GLP had already lost all its leaves.

### Pigment analysis

Total chlorophyll (Chl_TOT_), β-carotene and xanthophyll (violaxanthin, V; antheraxantin, A; zeaxanthin, Z) content were determined by HPLC (P680 HPLC Pump, UVD170U UV-Vis detector, Dionex, Sunnyvale, CA, USA) according to Döring et al. (2014). In order to measure the pigment content, a known quantity of pure standard was injected into the HPLC system and an equation, correlating the peak area to pigment concentration, was formulated. The data were processed using Dionex Chromeleon software. The de-epoxidation state of xanthophyll cycle (DES) was calculated as (A0.5+Z)/VAZ.

The anthocyanin content was estimated by using the Dualex Scientific optical sensor (Force-A, Centre Universitaire Paris Sud, France). It measures the leaf epidermal anthocyanin absorbance at 520 nm by means of the chlorophyll fluorescence screening method (Agati et al., 2011), equalizing the chlorophyll fluorescence signal under the 520 nm excitation and that under red excitation at 650 nm, as reported in Goulas et al. (2004).

### H_2_O_2_ and ${\text{O}}_{2}^{-}$ quantification

H_2_O_2_ content was determined using the Amplex Red Hydrogen Peroxide/Peroxidase Assay Kit (Molecular Probes, Invitrogen, Carlsbad, CA, USA) according to Pellegrini et al. (2013). Analyses were performed spectrometrically with a fluorescence/absorbance microplate reader (Victor3 1420 Multilabel Counter Perkin Elmer, Waltham, MA, USA). ${\text{O}}_{2}^{-}$ concentration was measured according to Tonelli et al. (2015) using a spectrophotometer (6505 UV-Vis, Jenway, Stone, UK).

### Confocal laser scanning microscopy and leaf anatomy

Leaf pieces of approximately 4 × 8 mm in size were cut with a razor blade and imaged using a Leica TCS SP8 confocal upright microscope (Leica Microsystems CMS, Mannheim, Germany) equipped with a × 63 objective (HC PL APO CS2 63 × 1.40 OIL). Anthocyanins were localized by their autofluorescence excited at 488 nm, through a DD 488/552 beam splitter and acquired over the 526–598 nm emission spectral band. Image spatial calibration was between 0.12 and 0.16 μm pixels^−1^. Free hand leaf cross section, were viewed under a microscope (Helmut Hund D-6330 Wetzlar, Germany) to measure the thickness.

### Statistical analyses

Physiological and biochemical data were analyzed by two-way ANOVA using genotype and sampling date as the variability factors followed by Fisher's least significant difference (LSD) *post-hoc* test (*P* = 0.05). Before the ANOVAs, the assumption of homogeneity of variances was tested using Bartlett's test. Nitrogen content was analyzed using Student's *t*-test with sampling data as the variability factor. Linear regression was used to determine the light mitochondrial respiration (R_d_), the CO_2_ compensation point in the absence of respiration Γ^*^, and the carboxylation efficiency of Rubisco, V_cmax_. Non-linear least square regression was used to interpolate the response of CO_2_ assimilation to light (light curves) or to Ci (A/Ci curves). Percentage data were angularly transformed prior to the statistical analysis. All statistical analyses were conducted using GraphPad (GraphPad, La Jolla, CA, USA).

## Results

### Photosynthetic parameters

**Figure 1:**
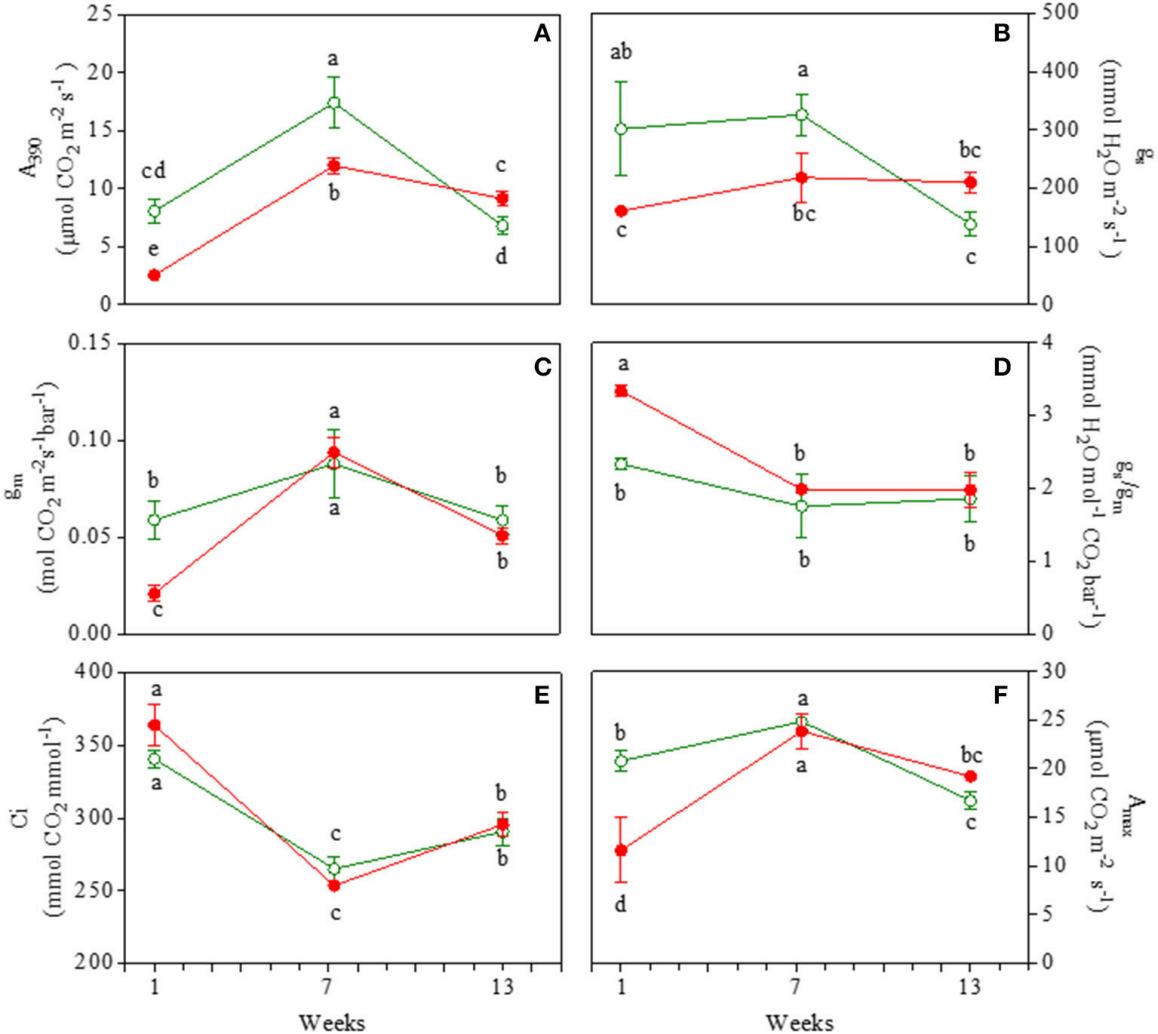
Net photosynthesis at saturating light and ambient CO_2_ (A_390_; **A**); stomatal conductance (g_s_; **B**); mesophyll conductance (g_m_; **C**), ratio of g_s_/g_m_ **(D)**, intercellular CO_2_ concentration (Ci; **E**), net photosynthesis at saturating light and CO_2_ concentration (A_max_; **F**) in 1-, 7-, and 13-week-old leaves of *Prunus cerasifera* clone 29C (open circles) and *Prunus cerasifera* var. *Pissardii* (closed circles). Means (±SD; *n* = 5) were compared by two-way ANOVA with morph and sampling date as sources of variation. Means flanked by the same letter are not statistically different for *P* = 0.05 after Fisher's least significant difference *post-hoc* test.

The net photosynthetic rate at ambient CO_2_ (A_390_) was 33% greater in juvenile GLP than RLP leaves (Figure 1A). Net photosynthesis increased in mature leaves of both morphs; the gap between the two morphs was similar to that found in the juvenile leaves. In 13-week-old leaves, A_390_ decreased more in GLP (−62%) than RLP (−25%) leaves, with RLP photosynthesizing more than GLP leaves (9.2 ± 0.6 and 6.8 ± 0.7, respectively). The gap of stomatal conductance (g_s_) between green and red leaves was consistent with A_390_ (Figure 1B). Mesophyll conductance (g_m_) was lower (−68%) in juvenile RLP than GLP leaves, whereas no differences between GLP and RLP were found in 7- and 13-week-old leaves (Figure 1C). This meant that RLP young leaves had a higher g_s_/g_m_, whereas no statistical differences were found during the ontogenesis between GLP and RLP (Figure 1D). No differences in intercellular CO_2_ concentration were observed between the two leaves upon leaf ontogenesis (Figure 1E). Finally, the photosynthetic rate in unlimited light and CO_2_ conditions (A_max_) was lower in red leaves 1 week after emergence (−56%), compared to green leaves. A_max_ reached similar values in RLP and GLP mature leaves, and decreased to a similar extent in 13-week-old leaves of both morphs (Figure 1F).

**Figure 2:**
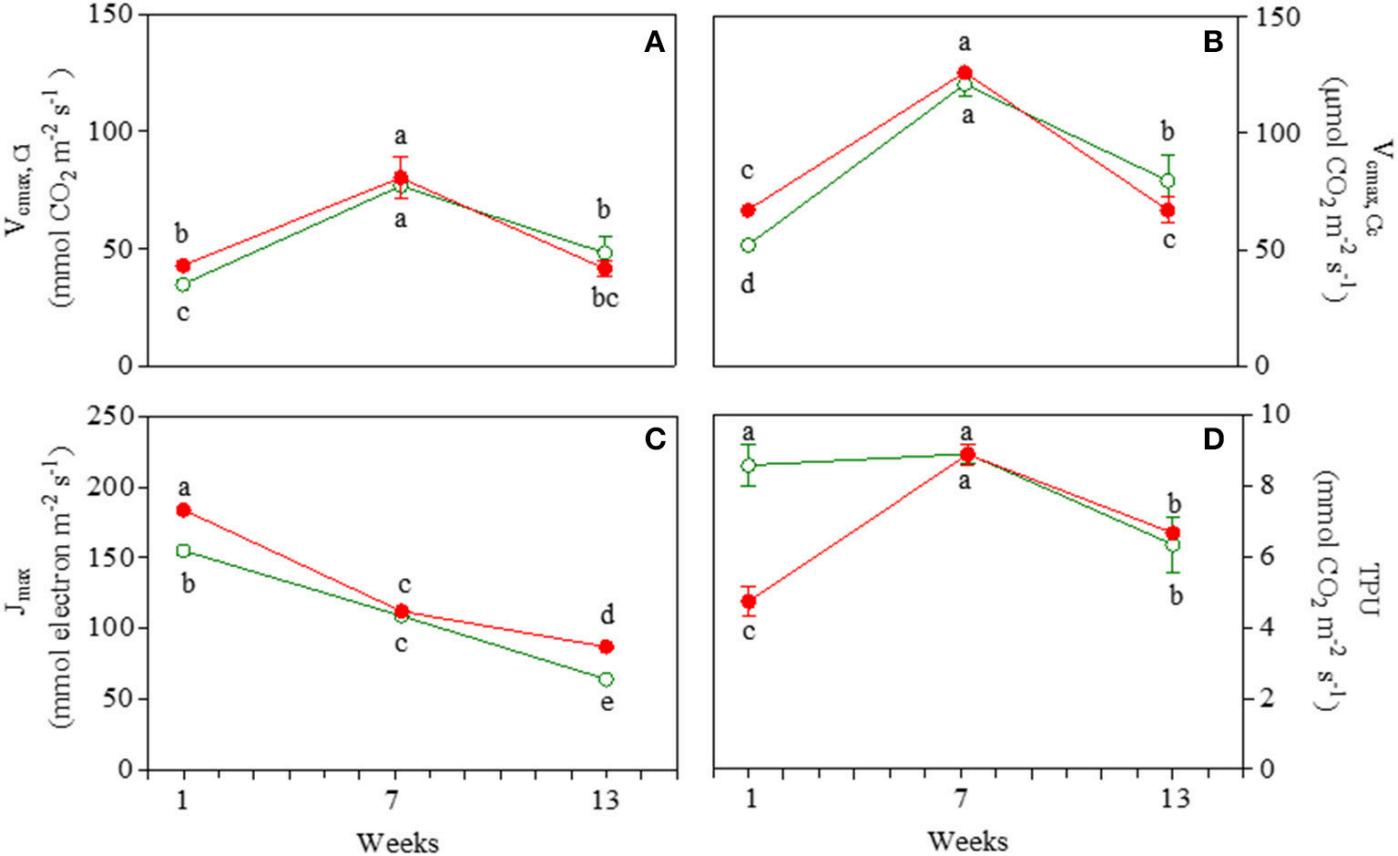
Values of apparent maximum rate of carboxylation by Rubisco at intercellular (V_cmax,Ci_; **A**) and chloroplastic (V_cmax,Cc_; **B**) CO_2_ concentration, maximum electron transport rate (J_max_; **C**), and triose phosphate utilization rate (TPU; **D**) in 1-, 7-, and 13-week-old leaves *Prunus cerasifera* clone 29C (open circles) and *Prunus cerasifera* var. *Pissardii* (closed circles). Means (±SD; *n* = 5) were compared by two-way ANOVA with morph and sampling date as sources of variation. Means flanked by the same letter are not statistically different for *P* = 0.05 after Fisher's least significant difference *post-hoc* test.

Morph and date sampling affected V_cmax_ at both chloroplastic and intercellular CO_2_ concentrations (Figures 2A,B; Table S1). Values of V_cmax_ were ~2-fold higher in mature leaves of GLP and RLP compared to 1- and 13-week-old leaves. Most GLP and RLP values overlapped upon ontogenesis. Note that V_cmax(Ci)_ and V_cmax(Cc)_ were higher in red than green juvenile leaves.

J_max_ decreased significantly during leaf ontogenesis (−59 and −53% in green and red leaves, respectively, when comparing 13- vs. 1-week-old leaves; Figure 2C) where (in both stages) higher values were recorded in red compared to green leaves. For both GLP and RLP, the pattern of TPU overlapped that of A_max_ throughout the experiment (Figure 2D).

### Soluble sugar, sorbitol, starch, and nitrogen content

**Figure 3:**
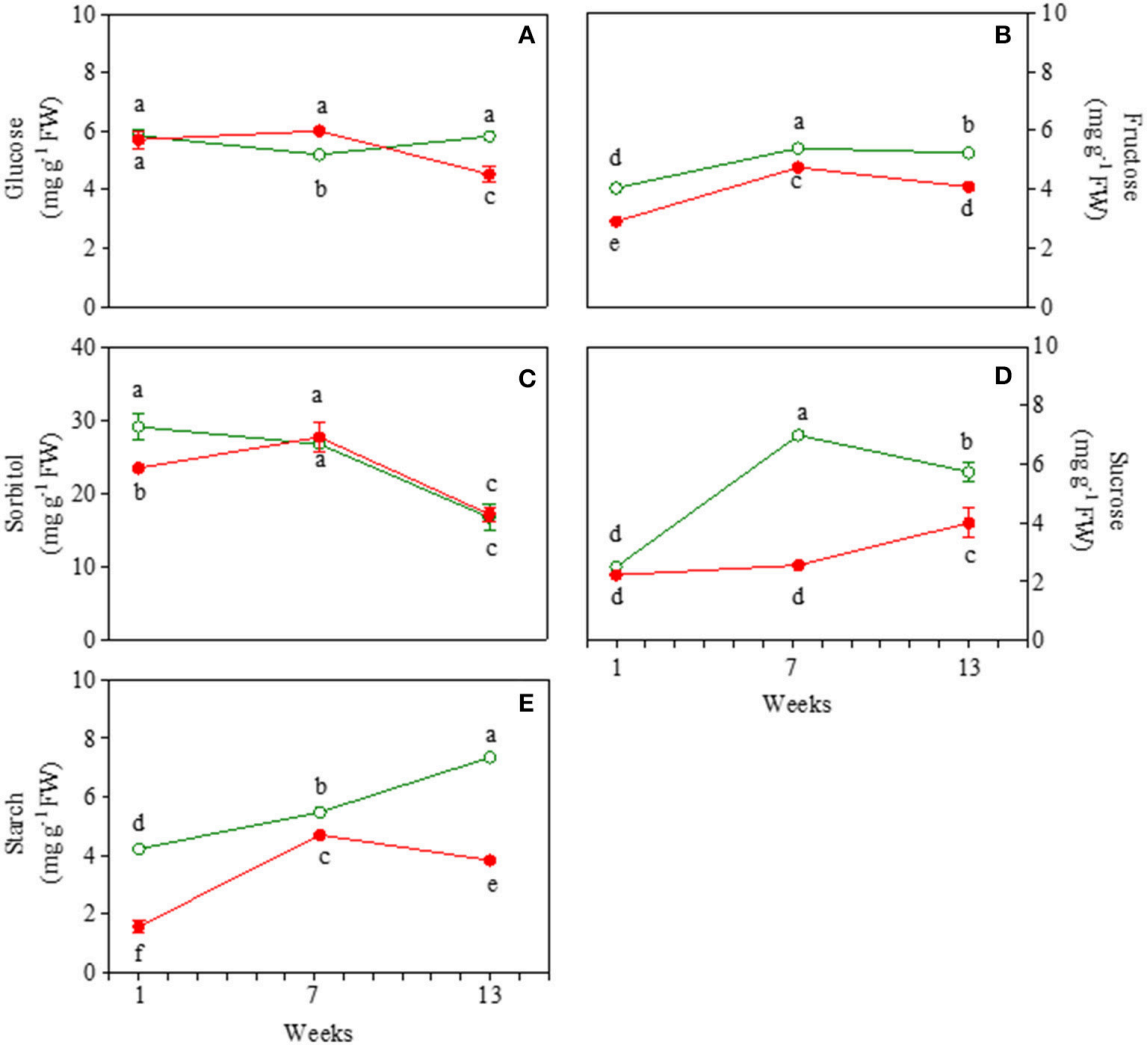
Glucose **(A)**, fructose **(B)**, sorbitol **(C)**, sucrose **(D)**, and starch **(E)** content 1-, 7-, and 13-week-old leaves of *Prunus cerasifera* clone 29C (open circles) and *Prunus cerasifera* var. *Pissardii* (closed circles). Means (±SD; *n* = 5) were compared by two-way ANOVA with morph and sampling date as sources of variation. Means flanked by the same letter are not statistically different for *P* = 0.05 after Fisher's least significant difference *post-hoc* test.

Glucose content was similar in juvenile green and red leaves, but showed a very different behavior in mature and early senescent leaves (Figure 3A). In mature leaves, glucose content decreased only in GLP. In senescent leaves, an increase in glucose in GLP and in turn a strong decrease (−31%) in red leaves was found. Fructose content was always higher in green than red leaves, irrespectively of the leaf stage (Figure 3B). It increased from young to mature leaves and then decreased slightly at the end of the experiment.

Sorbitol content was higher in young green then red leaves (Figure 3C). At 7 weeks it increased in red leaves reaching values close to green mature leaves, whose values did not change compared to the juvenile counterpart. A similar drop in sorbitol content (on average−36%) was found in 13-week-old leaves of both morphs. Sucrose content was similar in young leaves of both morphs and remained lower in red compared to green leaves, with only a slight increase at the end of the experiment (Figure 3D). In green leaves, a strong and significant increase in sucrose content was observed in 7-week-old leaves compared to young leaves, and unlike red ones at the end of the experiment there was a slight decrease. Starch content increased constantly upon leaf ontogenesis in GLP whereas, after a build-up also observed in RLP, a decline was found in 13-week-old leaves of RLP (Figure 3E). Starch content was always higher in green than red leaves, especially in young (3-fold) and senescent leaves (2.6-fold) (Figure 3E).

Leaf nitrogen content was similar in both 7- and 13-week-old leaves when comparing GLP and RLP (Table 1). However, 13-week-old leaves of RLP showed a sensitively lower N resorption efficiency compared to GLP (24.1 ± 1.3 vs. 42.7 ± 3.3, respectively). Only for red leaves, was N content measured in 17-week-old leaves, whereas at this stage, GLP had lost all its leaves (Table 1). In 17-week-old red leaves, N content was lower than 13-week-old GLP leaves, and we found a higher N resorption efficiency (55.3 ± 4.3) than in senescent green leaves (42.7 ± 3.3).

**Table 1:** Nitrogen content (g kg^−1^ DW) in leaves *Prunus cerasifera* clone 29C and *Prunus cerasifera* var. *pissardii* upon leaf ontogenesis starting 1 week after the leaf emergence.

	**Week 7**	**Week 13**	**N resorption efficiency (N_7_-N_13_)/N_7_ × 100**	**Week 17**	**N resorption efficiency (N_7_-N_17_)/N_7_ × 100**
*Prunus cerasifera* clone 29C	27.2 ± 0.1	15.6 ± 0.1	42.7 ± 3.3	–	
*Prunus cerasifera* var. *pissardii*	25.7 ± 0.6	19.5 ± <0.1.	24.1 ± 1.3	11.5 ± 0.2	55.3 ± 4.3
*P*	ns	ns	^***^		

Data were subjected to Student's t-test with genotype as variability factor. Data are means of 3 replicates ± S.D. In the last row the significance of the test is reported (ns: P > 0.05;

*** *P < 0.001). The N resorption efficiency is calculated as reported in Materials and Methods section*.

### Anthocyanin index and pattern of epidermal anthocyanins

**Figure 4:**
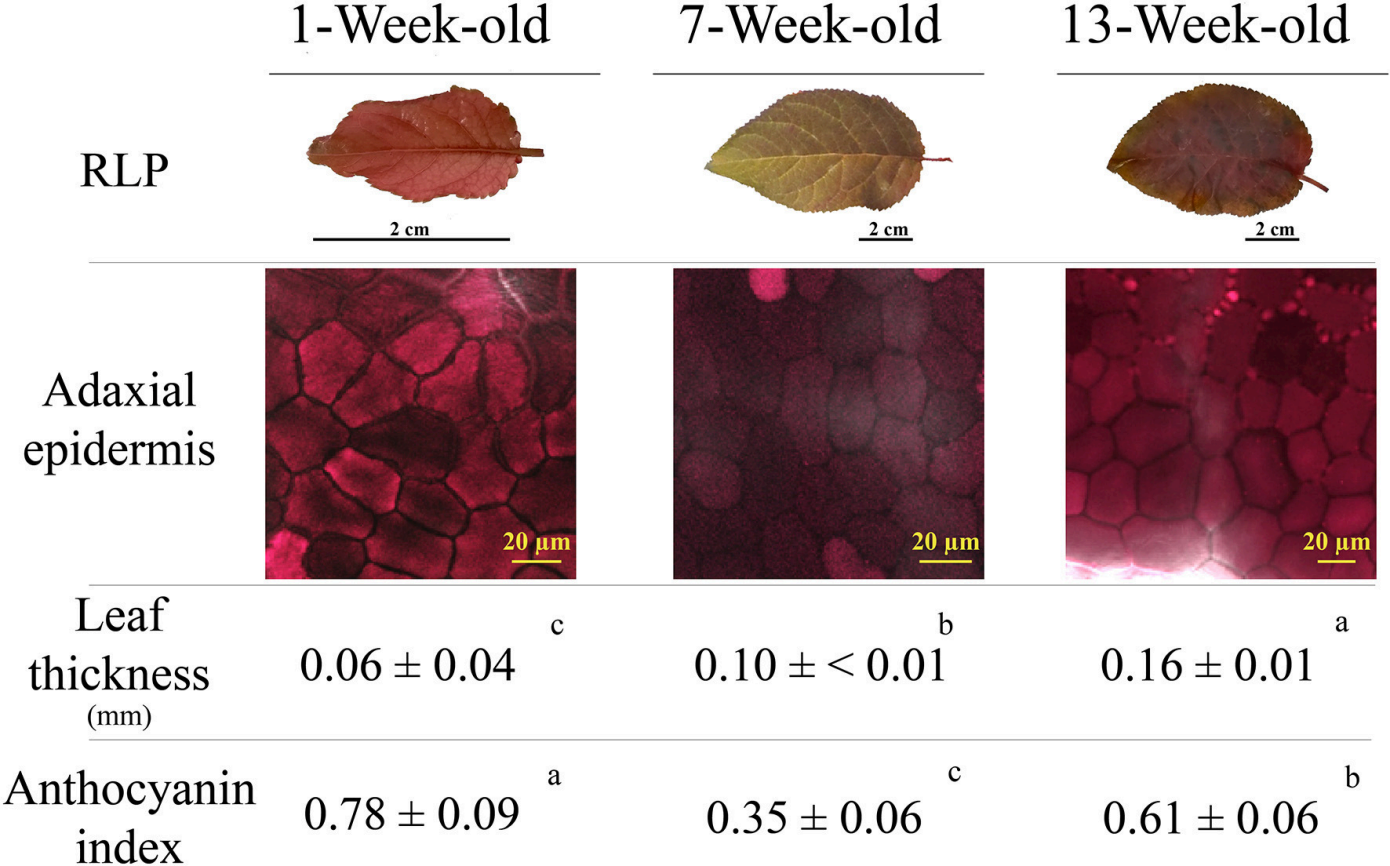
Leaf appearance, anthocyanin fluorescence signal over the adaxial epidermis (confocal microscope), leaf thickness, and anthocyanin index (see Materials and Methods section for the details) determined in 1-, 7-, and 13-week-old leaves of *Prunus cerasifera* var. *Pissardii*. Means (±SD; *n* = 5 and *n* = 10 for thickness and anthocyanin index, respectively) were compared by one-way ANOVA with sampling date as sources of variation. Means flanked by the same letter are not statistically different for *P* = 0.05 after Fisher's least significant difference *post-hoc* test.

Figure 4 reports various red leaf characteristics. A partial discoloration of mature RLP leaves was observed, which paralleled the decline in the anthocyanin index (from 0.78 to 0.35 in juvenile and mature leaves, respectively). Discoloration was due to the loss of anthocyanins in the upper epidermis, as highlighted by images obtained by confocal fluorescence microscopy (Figure 4). Differences upon ontogenesis were also detected in leaf thickness, which was higher in leaves 13 weeks after emergence compared to previous samplings. The anthocyanin index increased again in senescent leaves (0.61), but did not reach the values of juvenile leaves. A very low amount of anthocyanin was consistently present in green leaves throughout leaf ontogenesis (on average 0.15 ± 0.01; *data not shown*).

### Chlorophyll *a* fluorescence parameters

**Figure 5:**
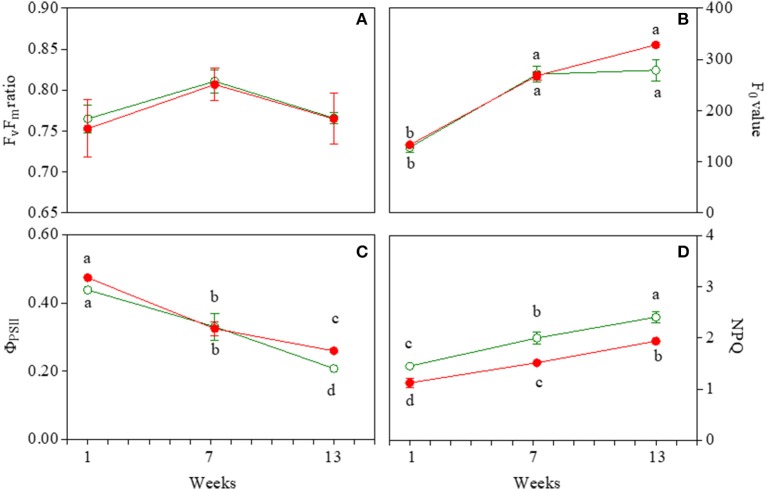
Photosystem II maximum photochemical efficiency (F_v_/F_m_; **A**), minimal fluorescence yield (F_0_; **B**), effective photochemical efficiency (φ_PSII_; **C**), and non-photochemical quenching (NPQ; **D**) in 1-, 7-, and 13-week-old leaves of *Prunus cerasifera* clone 29C and *Prunus cerasifera* var. *Pissardii*. Means (±SD; *n* = 5) were compared by two-way ANOVA with morph and sampling date as sources of variation. Means flanked by the same letter are not statistically different for *P* = 0.05 after Fisher's least significant difference *post-hoc* test. Lack of letters denotes non statistical significance of the interaction.

Effective (Φ_PSII_) and maximum (F_v_/F_m_) quantum yield of PSII varied in similar ways in both GLP and RLP leaves upon leaf ontogenesis (Figures 5A,C; Table S1). The only exception was the higher Φ_PSII_ of 13-week-old RLP leaves (Figure 5C). Young and early senescent leaves of both RLP and GLP were similarly photoinhibited (F_v_/F_m_ averaged 0.75 and 0.76, respectively), whereas both mature leaves had values typical of healthy plants (Figure 5A). The effective quantum yield decreased significantly upon leaf ontogenesis, with the lowest values recorded 13 weeks after emergence in both morphs (Figure 5C). The minimum fluorescence yield, F_0_, increased significantly upon leaf ontogenesis without differences in relation to morphs (Figure 5B). In young leaves, the minimum fluorescence was significantly lower than the other two leaf stages (Figure 5B; Table S1). NPQ values were always higher in GLP than RLP leaves and increased significantly and consistently during leaf ontogenesis (Figure 5D).

**Figure 6:**
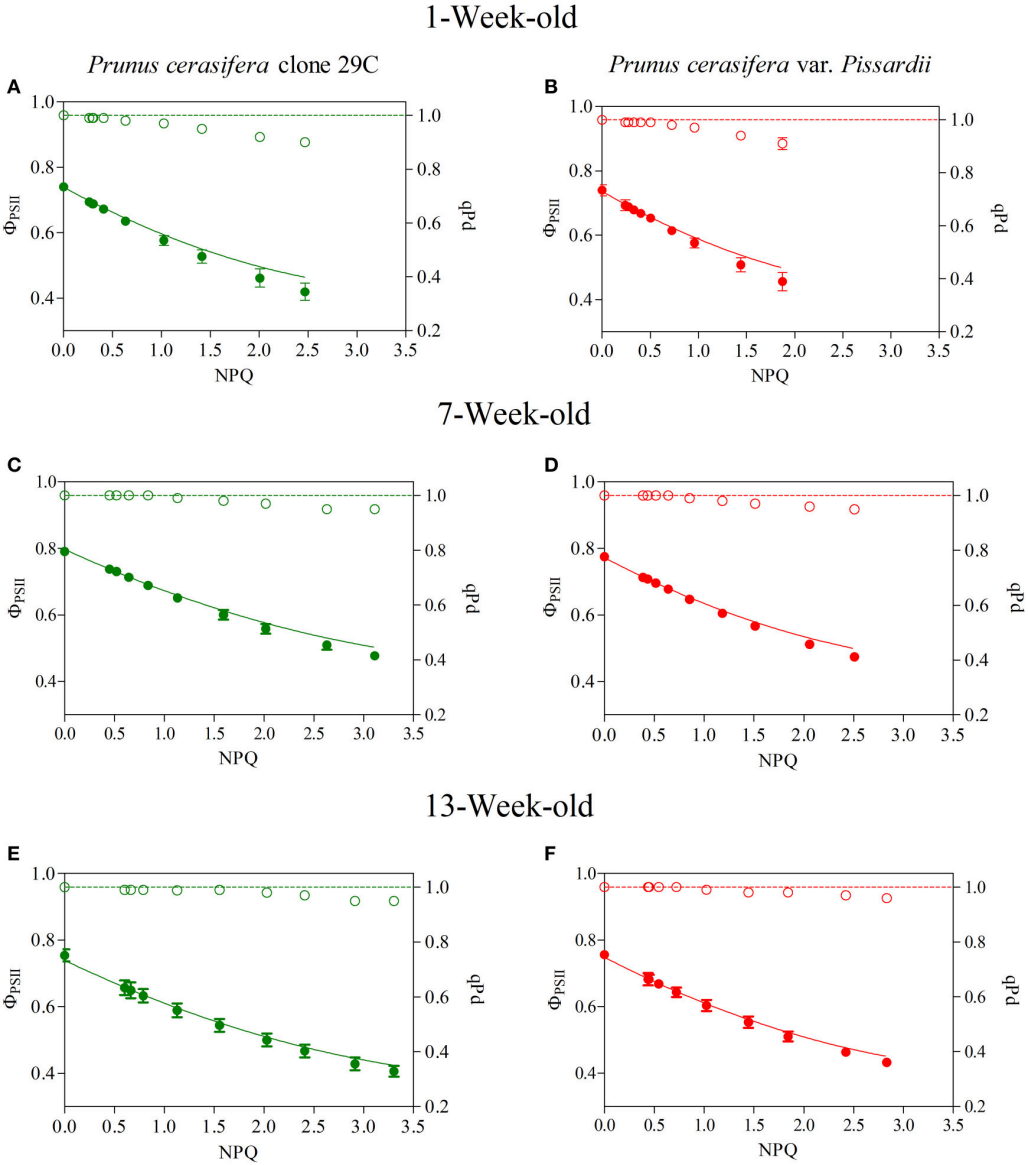
Relationship between NPQ and qPd (open circles) and NPQ and photosystem II effective quantum yield (closed circles) determined in 1-, 7-, and 13-week-old leaves of *Prunus cerasifera* clone 29C (**A,C,E**, respectively) and *Prunus cerasifera* var. *Pissardii* (**B,D,F**, respectively). Data are means of at least 12 replicates of intact leaves; error bars represent the standard error. Theoretical quantum yield (continuous line) was calculated using the Equation (1) reported I Materials and Methods section with qPd = 1.

The decrease in qPd, photochemical quenching in the dark, reflects the onset of photoinhibition, and we used an approach that assesses NPQ protection against photoinhibition of RCII, called protective NPQ or pNPQ (Ruban and Murchie, 2012). Figure 6 shows qPd and Φ_PSII_ changes in relation to NPQ determined during ontogenesis in the two morphs exposed to increasing light intensities (see Materials and Methods). In the absence of photoinhibition (i.e., qPd > 0.98), the actual Φ_PSII_ followed the same trend as the theoretical Φ_PSII_ (Figure 6), and decreased upon the NPQ gradient only because of the induction of pNPQ. Overall, the results obtained in the two morphs showed that up to high NPQ values, there was a good overlap between the experimental data and theoretical Φ_PSII_ upon NPQ, when qPd was close to 1. However, at the light intensity when qPd < 0.98, photoinhibition reduced the PSII photochemistry, leading to a discrepancy between actual and theoretical Φ_PSII_ (Figure 6). This effect was more pronounced in young leaves of both morphs in which the photoprotective capacity of NPQ decreased also for values of about 1 (Figures 6A,B). In mature leaves, the dynamics of qPd and NPQ differed from those exhibited by young leaves, and NPQ was protective against significantly higher light intensity (Figures 6C,D). Finally, in 13-week-old leaves, NPQ strongly increased reaching values close or even higher than 3, and photoinhibition was detected in both morphs only at a high light intensity (Figures 6E,F).

**Figure 7:**
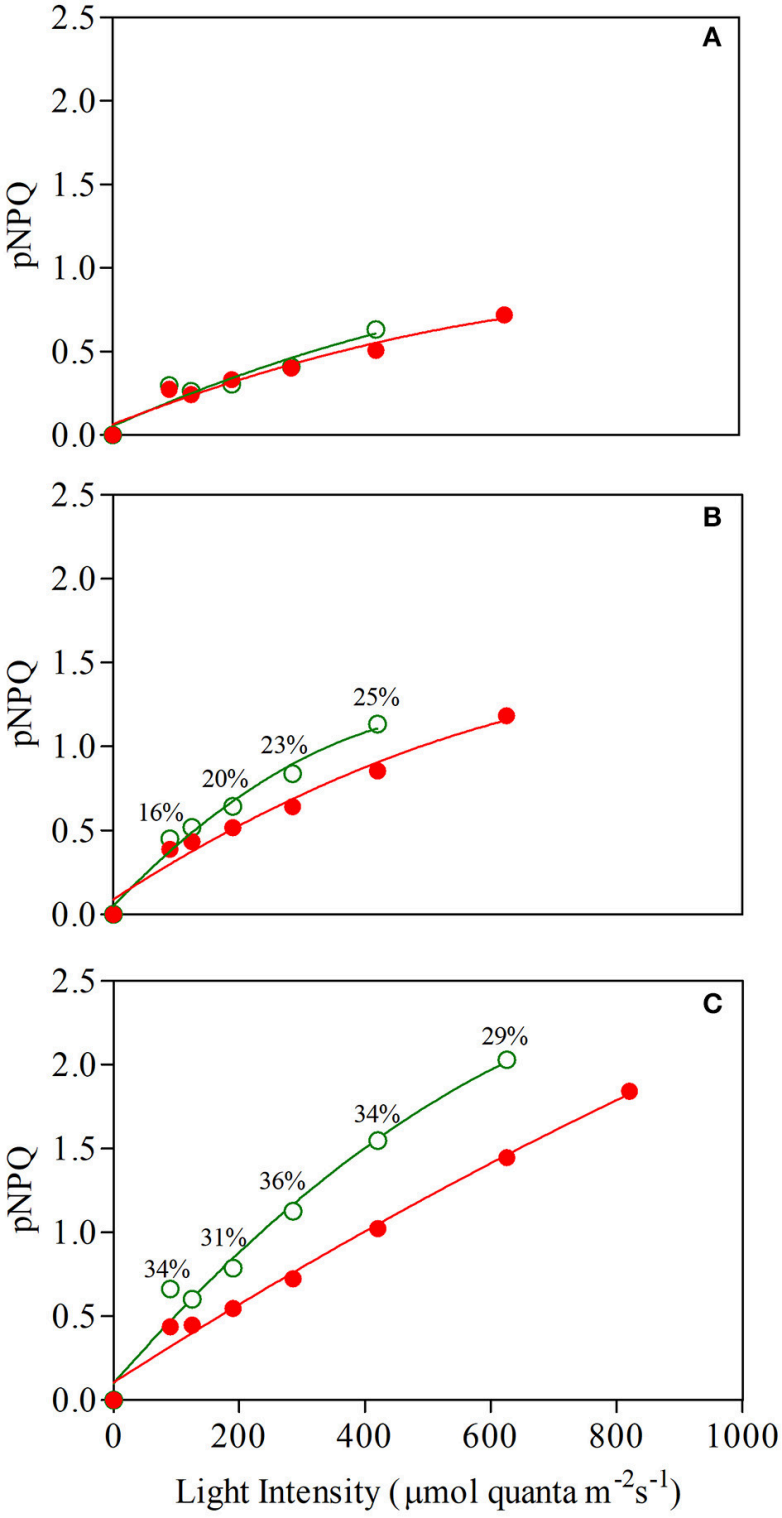
Relationship between light intensity and maximum photoprotective capacity (pNPQ) during a gradually increasing routine (see Materials and Methods for details) determined in 1- **(A)**, 7- **(B)**, and 13-week-old **(C)** leaves of *Prunus cerasifera* clone 29C (open circles) and *Prunus cerasifera* var. *Pissardii* (closed circles). Bars indicate % difference of pNPQ between red- and green-leafed *Prunus* under the same actinic light intensity.

Figure 7 shows the relationship between protective NPQ (pNPQ) and increasing actinic light. The curves represent the best fit of the lowest pNPQ point and indicate the minimum levels of pNPQ needed to protect PSII against photoinhibition at each intensity. GLP appeared to be less photoprotected than RLP juvenile leaves, which were not photoinhibited (qPd > 0.98) even at a higher light intensity (420 vs. 625 μmol m^−2^s^−1^, respectively; Student's *t*-test; *P*>0.01) (Figure 7A). This discrepancy between red and green leaves was consistent throughout the leaf ontogenesis.

In addition, there were dramatic differences between the NPQ values shown by both morphs at increasing light conditions. For example, both mature leaves were photoprotected at 420 μmol m^−2^s^−1^ (red leaves also at 625 μmol m^−2^ s^−1^; qPd was 0.985), but GLP had pNPQ values close to 1.1, whereas pNPQ of RLP was around 0.62 (Figure 7B). Again, similar differences in the level of pNPQ at increasing irradiances were found in senescent leaves, in which RLP leaves (but not GLP) were not photoinhibited also at 820 μmol m^−2^ s^−1^ and had −30% lower pNPQ at 625 μmol m^−2^ s^−1^ (Figure 7C).

### Pigment content

**Figure 8:**
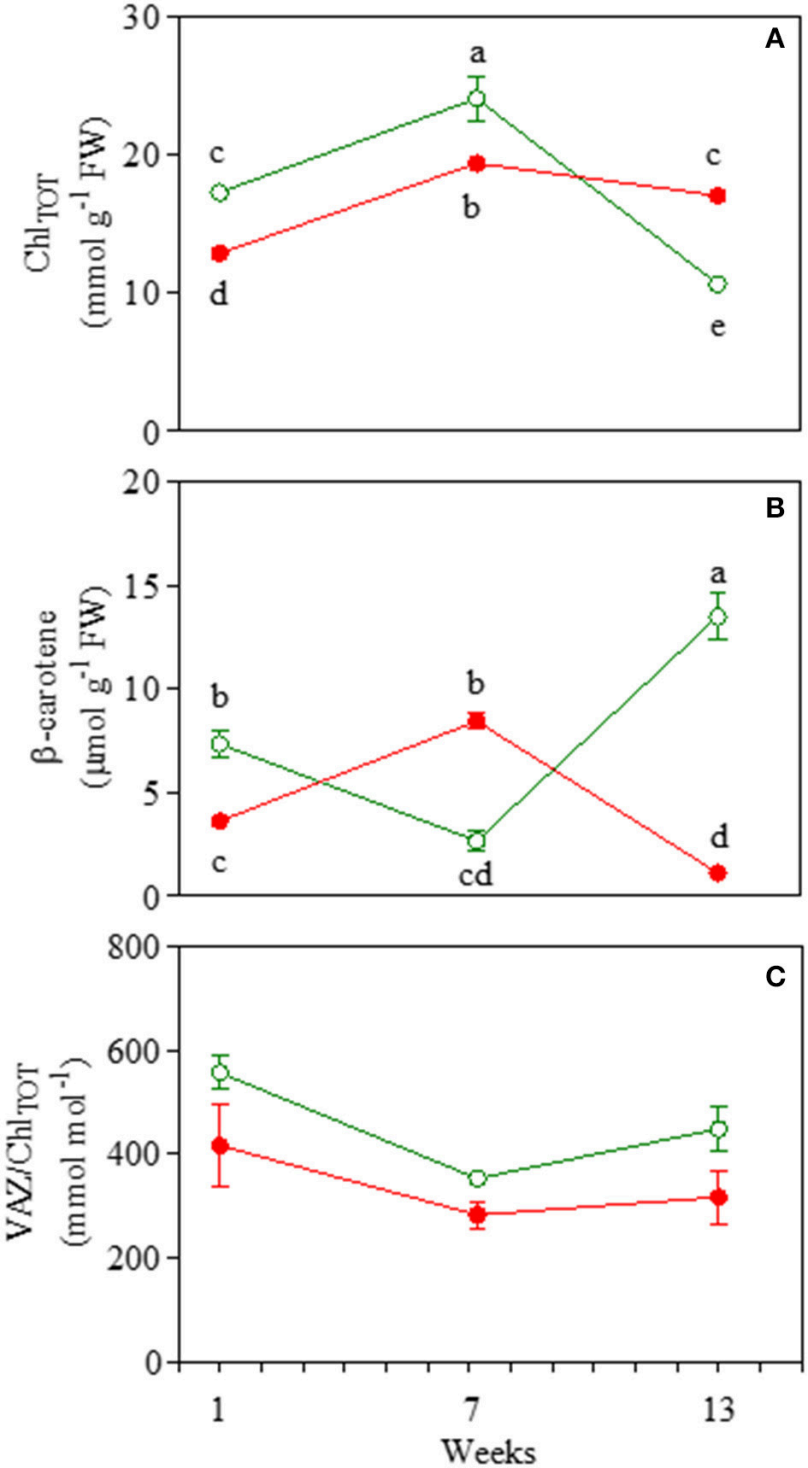
Total chlorophyll (Chl_TOT_; **A**), (β-carotene; **B**), and total xanthophyll (VAZ/Chl_TOT_; **C**) content in 1-, 7-, and 13-week-old leaves *Prunus cerasifera* clone 29C (open circles) and *Prunus cerasifera* var. *Pissardii* (closed circles). Means (±SD; *n* = 5) were compared by two-way ANOVA with morph and sampling date as sources of variation. Means flanked by the same letter are not statistically different for *P* = 0.05 after Fisher's least significant difference *post-hoc* test. VAZ indicates the sum of violaxanthin (V), antheraxanthin (A) and zeaxanthin (Z).

Total chlorophylls followed a typical leaf pattern during ontogenesis: they increased in mature leaves of both morphs and significantly decreased toward the end of the experiment (Figure 8A). Red leaves had a significantly lower concentration of Chl_TOT_ at 1 and 7 weeks compared to green leaves. However, at the end of the experiment, red leaves contained a much higher amount of total chlorophyll compared to green leaves (Figure 8A). Carotenoids varied greatly during leaf ontogenesis (Figures 8B,C). Interestingly, β-carotene increased strongly at the end of the experiment in green leaves when a strong decrease in total chlorophyll was detected (Figure 8B). In red leaves however, β-carotene increased in red mature leaves when epidermal anthocyanin strongly decreased (Figure 4, 8B). Concentrations of VAZ/Chl_TOT_ were always lower in RLP than GLP (Figure 8C). In both RLP and GLP mature leaves, VAZ/Chl_TOT_ followed the same decline, whilst in 13-week-old leaves, the ratio remained unchanged in RLP and increased again in GLP.

### H_2_O_2_ and ${\text{O}}_{2}^{-}$ levels

**Figure 9:**
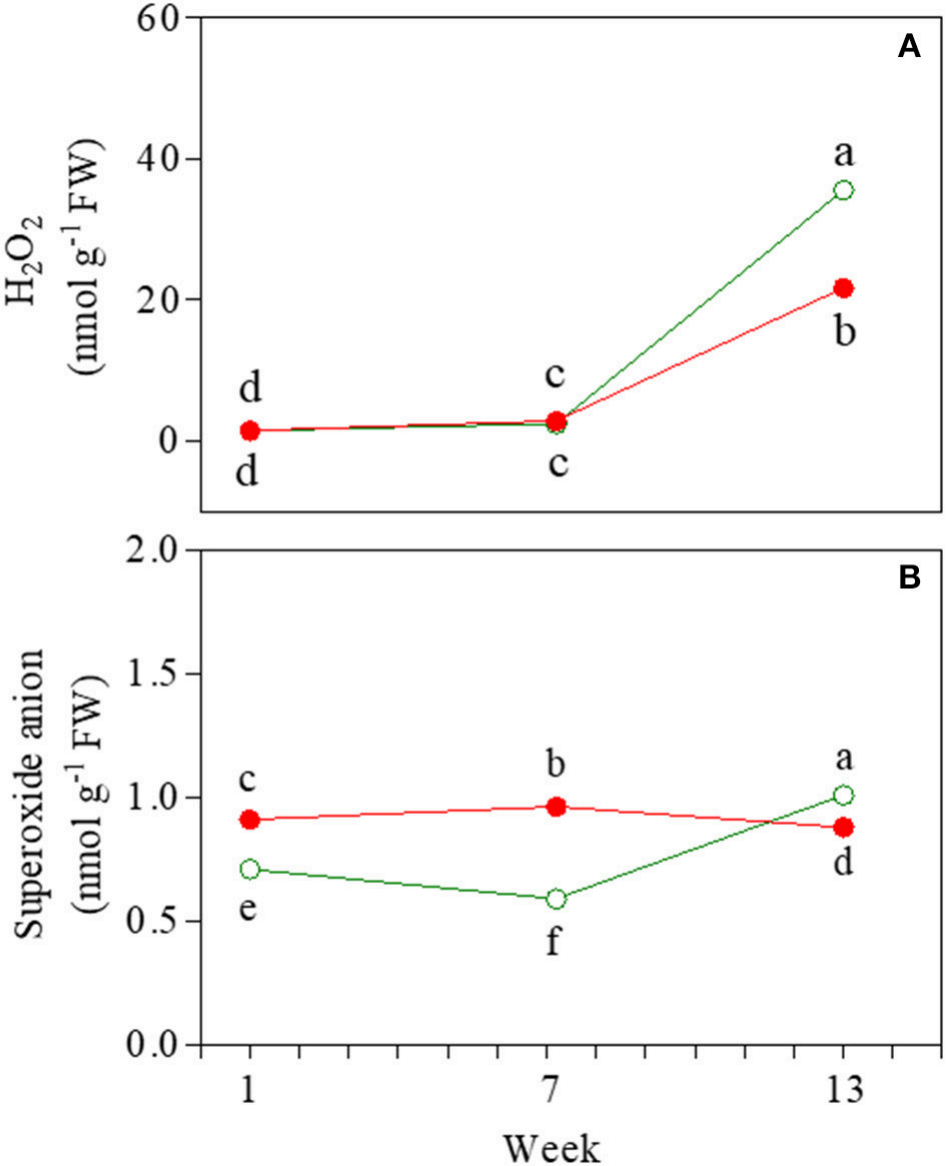
Hydrogen peroxide **(A)** and superoxide anion **(B)** content determined in 1-, 7-, and 13-week-old leaves of *Prunus cerasifera* clone 29C (open circles) and *Prunus cerasifera* var. *Pissardii* (closed circles). Means (±SD; *n* = 3) were compared by two-way ANOVA with morph and sampling date as sources of variation. Means flanked by the same letter are not statistically different for *P* = 0.05 after Fisher's least significant difference *post-hoc* test.

H_2_O_2_ and ${\text{O}}_{2}^{-}$ were detected as markers of oxidative stress during ontogenesis. The amount of H_2_O_2_ was lower in red than in green senescent leaves (21.7 and 35.6 nmol g^−1^ FW, respectively), whereas no differences were found in either juvenile or mature leaves (Figure 9A). Higher values of ${\text{O}}_{2}^{-}$ contents were also detected in senescent green leaves (Figure 9B).

## Discussion

### Photosynthesis of red and green leaves during ontogenesis

The attenuation of a proportion of green-blue wavebands reaching the leaves, as well as the metabolic cost associated with the biosynthesis of anthocyanins, have been proposed as the dual reason for the inferior photosynthetic capacity often found in anthocyanin-equipped leaves (reviewed by Gould et al., 2009). In other cases, when the ability of anthocyanins to photoprotect the photosynthetic apparatus exceeds the apparent “disadvantage of being red,” anthocyanin-enriched leaves perform better than anthocyanin-less leaves (Gould et al., 1995).

Our study shows that mature leaves of RLP had a ~30% lower photosynthetic rate compared to GLP, in accordance with previous results in the same species (Kyparissis et al., 2007). A higher photosynthetic rate was also evident in juvenile leaves of GLP. In both, juvenile and mature GLP leaves, higher values of A_390_ paralleled to higher values of g_s_ compared to the respective RLP leaves. Stomatal conductance appeared to be the main photosynthetic limitation in mature leaves of RLP, since A_max_, V_cmax_, J_max_, and TPU are similar to those of mature GLP leaves. In juvenile RLP leaves, other biochemical limitations were also responsible for reduced levels of net photosynthesis, i.e., lower values of A_max_, V_cmax_, J_max_, and TPU, which indicates a slower development of functional chloroplasts than GLP (Hughes et al., 2007).

Irrespectively of the abatement of a proportion of blue photons reaching the antennae, the ability of anthocyanins to absorb blue light, the key driver of stomatal opening, may have further contributed to the lower photosynthesis of RLP than GLP juvenile and mature leaves (Kim et al., 2004; Talbott et al., 2006; Aasamaa and Aphalo, 2016). Interestingly, the stronger the accumulation of anthocyanin (juvenile vs. mature RLP), the more the stomata limitations we observed. The phrase “*Don't ignore the green light*” (Smith et al., 2017) stresses how the importance of green light for photosynthesis is usually overemphasized because of the misconception that plants poorly utilize green light, which is related to the near-ubiquitous green appearance (thus green reflectance) of plants on earth. Results obtained in 1- and 7-week-old RLP leaves confirm that the attenuation of a proportion of blue and (principally) green light induced the “shade acclimation syndrome” (Kyparissis et al., 2007), where mature RLP leaves exhibit altered morpho-anatomical (i.e., thinner leaves), physiological (i.e., lower values of g_s_), and biochemical traits (i.e., lower Chl *a*/*b, data not shown*) than GLP leaves. The strongest accumulation of epidermal anthocyanins found in juvenile RLP leaves paralleled the lowest values of g_m_, which (together with g_s_) is another limitation of CO_2_ diffusion typical of shaded plants (Terashima et al., 2009). However, in terms of light quality, the “shade acclimation syndrome” (green-depleted red-enriched shading) differs from natural shade, a condition of blue-depleted far-red-enriched light (Smith et al., 2017). In RLP, this explains the lack of some traits typical of shade leaves, such as similar values of g_m_ found in mature leaves of RLP and GLP, and (slightly) lower values of Chl_TOT_ in leaves of RLP than GLP. The disappearance of anthocyanins over the adaxial epidermis of mature RLP leaves may have weakened the shading effect of anthocyanins (detailed later).

The abovementioned results in young and mature leaves support the hypothesis that anthocyanic leaves are unable to cope with green counterparts in terms of photosynthetic rate (Hughes et al., 2014), given that on a long-term basis, the contribution to the overall carbon fixation of senescent leaves is usually less important.

To describe the full picture of the whole 13-week-period of leaf ontogenesis, however, some particular aspects of our experiments need to be considered. First, for the sake of simplicity in this report we only describe the pattern of the main photosynthetic parameters at midday, when green leaves doubtless performed better than red ones. Conversely, values of A_390_ in the early morning, in the afternoon and early in the evening did not differ between mature leaves of RLP and GLP (Figure S2). Second, 13-week-old leaves of RLP had about a +26% of net photosynthesis compared to those of GLP, with only a slight decline with respect to their relative 7-week-old leaves. Third, although our experiment stopped 13 weeks after leaf emergence, we noticed that the leaf lifespan of RLP was dramatically longer than that of GLP; RLP plants continued to maintain their leaves even 25–30 days after GLP leaves had completely fallen (Figure S3). The contribution of senescent leaves to the cumulative carbon gain might therefore have had a serious impact on RLP.

To describe the mechanisms underlying the longer leaf lifespan of RLP, the pattern of anthocyanin biosynthesis and carbon allocation throughout the whole leaf ontogenesis are discussed.

### Anthocyanins during leaf ontogenesis: when, where, and why?

The function(s) of foliar anthocyanins still puzzles plant ecologists and physiologists (Lev-Yadun and Holopainen, 2009; Hughes and Lev-Yadun, 2015; Landi et al., 2015). Today, the most accepted but still debated hypothesis is that these pigments may act as an efficient sunscreen, especially when anthocyanins are located in the leaf epidermis (review by Gould et al., 2018).

In RLP, anthocyanins are located in adaxial and abaxial leaf epidermis, mesophyll, and parenchymatic cells, and their accumulation parallels the higher need for photoprotection usually displayed by young and early senescent leaves (Merzlyak et al., 2008). A superficial interpretation of NPQ and VAZ/Chl values found in *Prunus* plants, which are always lower in red vs. green leaves, suggests that anthocyanins might be compensatory for xanthophylls in photoprotecting the leaf during leaf ontogenesis, which has been commonly observed in other species (Manetas et al., 2002; Hughes et al., 2012; Tattini et al., 2014, 2017). In addition, at each developmental stage, the fact that GLP leaves need higher values of NPQ to maintain similar levels of Φ_PSII_ and qPd to those of RLP supports this interpretation. A more extensive analysis of our full dataset reveals a more complex situation in which the comparable efficiency and functionality of PSII between RLP and GLP upon leaf ontogenesis requires the close interplay between anthocyanins and carotenoids in red leaves.

Young leaves of RLP and GLP had similar values of pNPQ at low irradiance, but RLPs were not photoinhibited (qPd > 0.98), even when supplied with over 50% of irradiances. Young RLP leaves showed the highest anthocyanin accumulation upon ontogenesis, suggesting that the synergic photoprotective function of both xanthophyll and anthocyanins is necessary for RLP to preserve photosystem II from excess light and to assist the development of functioning chloroplasts (Manetas et al., 2002). Besides the biochemical differences between red and green young leaves, both had similar levels of H_2_O_2_, which were similar to those of mature leaves. In addition, ${\text{O}}_{2}^{-}$ values in young RLP were not higher than those of mature red leaves.

The progressive development of functional chloroplasts from juvenile to mature leaves leads to increased levels of Chl_TOT_, whereas the xanthophyll pool remains almost constant, thus inducing a proportionate decline in the VAZ/Chl ratio (Bertamini and Nedunchezhian, 2003). Accordingly, in both red and green mature leaves, we found a similar increase in Chl_TOT_; however in mature RLP, the decline of VAZ/Chl was less proportionate than in GLP, and a higher availability of β-carotene, the direct precursor of xanthophylls, was also found. This paralleled the partial discoloration of the adaxial epidermis of mature RLP leaves, suggesting, again, a strict, well-orchestrated synergism between xanthophylls and anthocyanins to protect RLP leaves. This dynamic interplay obviated the need for a deep, permanent filter over the leaf, which would be disadvantageous in terms of light abatement under non-excessive light conditions. It also meant that RLP is more photoprotected, i.e., lower values of pNPQ and qPd>98 at higher irradiances. It is possible that the strong increment in β-carotene found in RLP might be a further necessary adjustment to protect weakly anthocyanin-screened leaves from supernumerary ROS generated by very quick transitory changes in light conditions (sunflecks) in which the dynamic photoprotective mechanism may fail to protect the photosynthetic apparatus efficiently (Pospíšil, 2012; Telfer, 2014). Accordingly, similar levels of ROS were recorded in both GLP and RLP at midday.

Thirteen-week-old red and green leaves exhibited typical symptoms of early senescence as revealed by changes in pigment composition and decline in photosynthetic performance deriving from the dismantling of chloroplasts (Abreu and Munné-Bosch, 2009; Schippers et al., 2015). For RLP leaves, in which the loss of chlorophyll was not as severe as that of GLP, dismantling occurred alongside a *de-novo* biosynthesis of anthocyanins over the adaxial epidermis, whose levels were comparable to those of young leaves. Conversely, VAZ/Chl values remained similar to those of mature leaves, whereas the level of VAZ/Chl in GLP increased during ontogenesis from the 7th to the 13th week. Similarly to the mature leaves, lower values of pNPQ (−32% on average) were found in red than green leaves, especially at high irradiances, thus indicating the anthocyanins' photoprotective role. On the other hand, the huge increase in β-carotene did not prevent green leaves from accumulating higher levels of H_2_O_2_ and ${\text{O}}_{2}^{-}$ compared to red leaves which suggests a minor (or null) influence of this compound as photo-protector in green leaves

Anthocyanin patterns during the leaf ontogenesis of *Prunus* confirm that these pigments assist xanthophylls in photoprotecting the leaves from excessive light, despite dynamic changes at the various leaf development stages. However, because anthocyanins accumulate strongly in young and senescent leaves, when there is both the need for photoprotection and to maintain a high carbon sink strength, it seems reasonable to ask whether photoprotection is the primary role of foliar anthocyanins.

### Interplay between anthocyanin, sugar metabolism, and leaf lifespan

There is an apparent inextricable link between the sunscreen effect of anthocyanins and the possibility that anthocyanins represent a carbon sink to buffer sugar levels when the rate of allocation of the newly synthesized carbon skeletons proceeds slower than that of photosynthesis, at least before some regulatory feedback mechanisms take place. However, it is surprising that there has been so much research into testing their photoprotective role, whereas few papers have proposed that anthocyanin biosynthesis is principally devoted to preventing transitory sugar accumulation under unfavorable conditions for the leaf.

**Figure 10:**
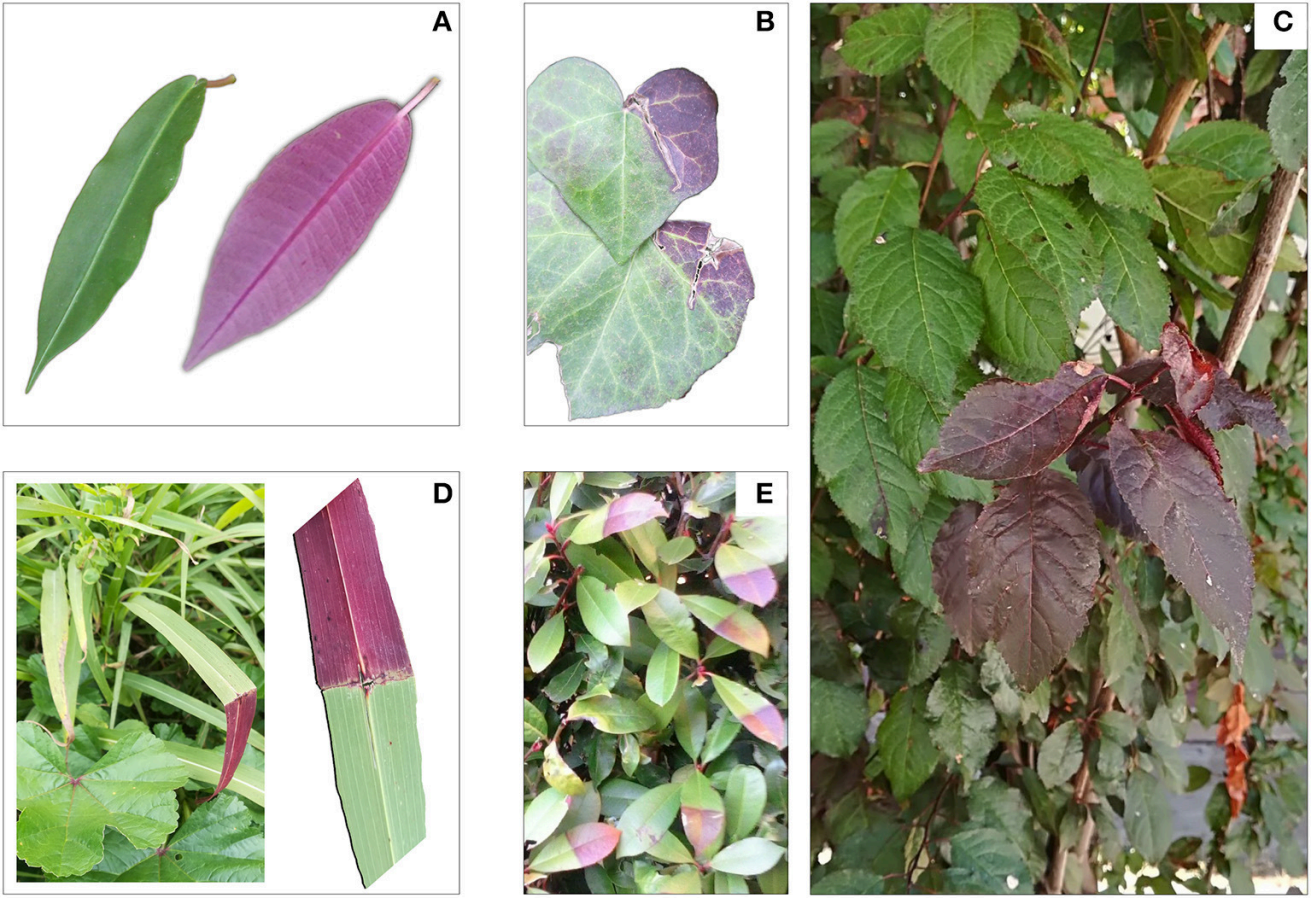
Adaxial (green) and abaxial (purple-red) side of leaves of *Fockea natalensis* **(A)**; effect of mechanical damage in leaves of *Hedera helix* **(B)**; effect of girdling in leaves of *Prunus cerasifera* var. *Pissardii* (red) grown at the bottom of the canopy **(C)**; Effect of disruption of the central vein in a leaf of *Hordeum vulgare* **(D)**; effect of girdling of the central vein of *Photonia x fraseri* “Red Rubin” **(E)**.

There are several factors indicating that photoprotection may not be the primary function of anthocyanins in leaves (some evidence is reported in Figure 10). First, around 30% of papers have failed to reveal a photoprotective role of these pigments (Gould et al., 2018). Second, in some cases, why do anthocyanins accumulate in both the leaf epidermis (such as in our *Prunus* plants; the effect of girdling in shade leaves; Figure 10C), or even only in the abaxial epidermis in species grown under shade (Figure 10A)? Third, we noted that girdling in the central vein of mature, green leaves of anthocyanin-producing species (Figures 10D,E), or accidental damage to the leaves of *Hedera helix* (Figure 10B) led these leaves to produce anthocyanins only in the distal part of the leaves from where the phloem was disrupted (and where sugars presumably accumulate), despite the fact that all the leaf surface experiences the same irradiance. Finally, the high amount of sugars (e.g., under high light conditions) has only been reported to promote the cellular biosynthesis of anthocyanins in some cases, which is the most visible result to the human eye, whereas in many other circumstances, different C-based secondary metabolites, with (Lichtenthaler et al., 2007), or without photoprotective functions (see for example Coley et al., 1985) have accumulated in the leaves. Accordingly, other authors have also suggested that phenolics, including anthocyanins, may be a carbon sink for absorbing excess photosynthetic carbon (Waterman et al., 1984).

However, there is much evidence that the reduction in sink strength induces sugar accumulation in the leaves followed by anthocyanin accumulation (Weiss, 2000; Hughes et al., 2005; Teng, 2005; Solfanelli, 2006; Peng et al., 2007, 2008; Murakami et al., 2008). The biosynthesis of anthocyanins in young leaves can sustain an active phloem flux of translocating sugars or polyols (such as sorbitol in our case) from source leaves, whereas anthocyanin biosynthesis within old leaves might present a means to moderate sugar feedback regulation, thereby avoiding the effect of early sugar-induced senescence. In fact, in our experiment we found the highest concentration of anthocyanins in young and early senescent leaves of RLP; in the latter there was also a build-up of sucrose, a strong anthocyanin-promoting agent (Hara et al., 2003; Teng, 2005; Solfanelli, 2006; Murakami et al., 2008). Increases in glucose, fructose, and starch found only in GLP leaves suggests advanced senescence in green rather than red leaves. Higher N resorption found in 17-week-old leaves of RLP (when GLP leaves had already fallen) compared to 13-week-old leaves of GLP is additional proof of the delayed senescence, which occurred in leaves of RLP.

**Figure 11:**
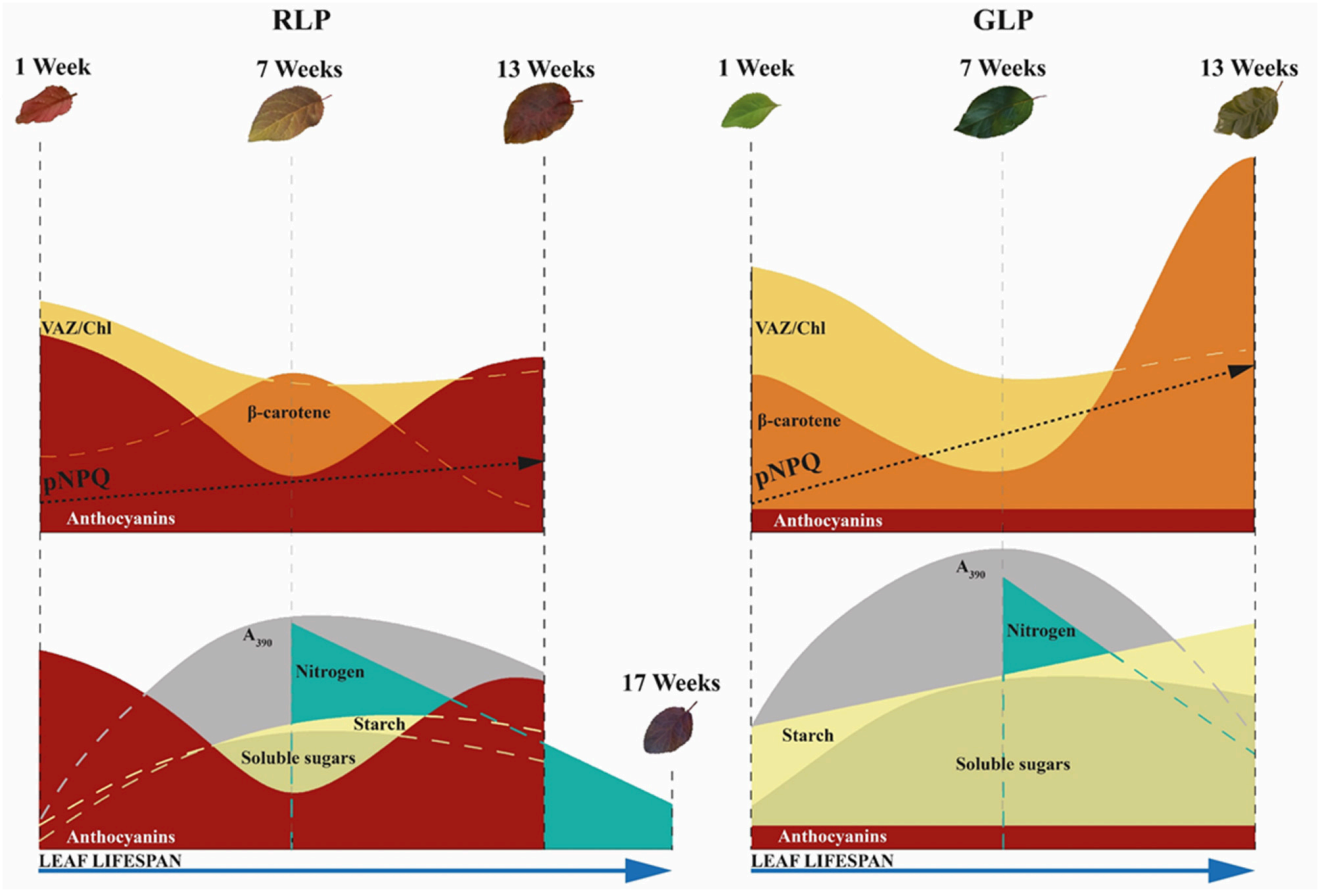
Variation in photoprotective mechanisms between green-leafed *Prunus cerasifera* clone 29C (GLP) and red-leafed *Prunus cerasifera* var. *Pissardii* (RLP) at different leaf stages (upper side). Variations in sugar, starch, and nitrogen remobilization in relation to respective photosynthetic capacity and leaf lifespan of GLP and RLP leaves upon leaf ontogenesis (bottom). pNPQ, maximum photoprotective capacity; VAZ, sum of violaxanthin (V), antheraxanthin (A) and zeaxanthin (Z); A_390_ net photosynthetic rate at saturating light and ambient CO_2_.

As schematized in Figure 11, the accumulation of anthocyanins in older RLP leaves may therefore (i) delay the sugar-promoted leaf senescence, (ii) maintain a more efficient photosynthetic apparatus for longer, and (iii) allow red leaves to translocate more N than GLP leaves, as already reported by previous research (Feild et al., 2001; Hoch et al., 2003). The capacity of anthocyanins to retard leaf senescence, thereby extending the leaf lifespan suggests the “conservative-use strategy” adopted by species with “long-lived organs” (e.g., evergreens) which inhabit nutrient-limiting environments in which a slower turnover of plant organs is advantageous (Valladares et al., 2000). When a RLP leaf abscises, it carries with it less than half of its maximum N (45%), as found in other conservative species (Chapin, 1980). On the other hand, GLP behaves like a fast-growing species that maximizes the biomass yield during favorable conditions and for which the loss of a higher level of N by leaf fall (57% was not recycled) can be easily compensated for by enhanced uptake mechanisms from soil in the following growing season.

The observations above do not deny that anthocyanins may have protective function. However, it seems more realistic that their photoprotective role, among others proposed regarding the plant-environment interaction (Archetti et al., 2009; Hughes and Lev-Yadun, 2015; Landi et al., 2015; Menzies et al., 2016), may only be a secondary adaptation occurring in the metabolism of plant species in which anthocyanins are transitory or permanently accumulated.

In conclusion, we wish to suggest the possibility that foliar anthocyanins firstly accumulate to prevent temporary sugar excess in source organs but, in turn, this implicates other biochemical and physiological consequences. Anthocyanin biosynthesis induces, for example, a downregulation of alternative mechanisms devoted to photoprotection (i.e., xanthophyll pool and/or antioxidant apparatus). So that, it is at least possible that there was a non-light-driven selective pressure of this pathway in ancestral species when the biosynthetic pathway of anthocyanins firstly evolved, but rather other factors connected to the sugar metabolism did. This is little more than a hypothesis, but we believe it warrants further study.

## Author contributions

LG, CN, RM, and DR designed the experiments. LG, ELP, and ML executed experiments and EP provided HPLC analysis. GA, and CG provided confocal microscope analyses. LG, ELP, ML, and EP analyzed results. LG, ELP, and ML, discussed results and conclusions of the study. LG, ELP, and ML wrote the manuscript. TG, GL, FM, CN, GR, and PV edited manuscript drafts. LG, RM, and GL provided funds for the experiments.

### Conflict of interest statement

The authors declare that the research was conducted in the absence of any commercial or financial relationships that could be construed as a potential conflict of interest.
