# Supplementary material for: Multiple Consequences Induced by Epidermally-Located Anthocyanins in Young, Mature and Senescent Leaves of Prunus
Source: Front Plant Sci. 2018 Jul 2;9:917. doi: 10.3389/fpls.2018.00917 (PMC6036500; doi:10.3389/fpls.2018.00917)
Supplement: Supplementary file 1 [file Table_1.DOCX]

**TABLE S1 ⎪ Value of F ratio and significance of different determined parameters following the two-way ANOVA test with morph and time as sources of variation. ***: P<0.001; **: P<0.01; *: P<0.05; ns: P>0.05).**

| Parameter | F value | P |
| --- | --- | --- |
| A_390_  Morph  Time  Morph*x*Time | 34.2  130.6  28.5 | ***  ***  *** |
| g_s_  Morph  Time  Morph*x*Time | 11.5  10.7  14.5 | **  **  *** |
| g_m_  Morph  Time  Morph*x*Time | 8.5  43.8  8.0 | *  ***  ** |
| g_s_/g_m_  Morph  Time  Morph*x*Time | 14.8  28.9  5.6 | **  ***  * |
| Ci  Morph  Time  Morph*x*Time | 1.8  16  5.6 | ns  ***  * |
| A_max_  Morph  Time  Morph*x*Time | 10.2  38.2  3.7 | **  ***  * |
| Vcmax_ci_  Morph  Time  Morph*x*Time | 0.5  118.1  18.8 | ns  ***  *** |
| Vcmax_cc_  Morph  Time  Morph*x*Time | 0.9  216.2  9.3 | ns  ***  ** |
| Jmax  Morph  Time  Morph*x*Time | 9040.1  1021.1  180.8 | ***  ***  *** |
| TPU  Morph  Time  Morph*x*Time | 56.7  98.3  73.6. | ***  ***  *** |
| Glucose  Morph  Time  Morph*x*Time | 4.9  14.3  41.8 | *  ***  *** |
| Fructose  Morph  Time  Morph*x*Time | 871.1  883.8  24.7 | ***  ***  *** |
| Sorbitol  Morph  Time  Morph*x*Time | 3.9  82.6  8.7 | **  ns  *** |
| Sucrose  Morph  Time  Morph*x*Time | 339.0  196.7  109.6 | ***  ***  *** |
| Starch  Morph  Time  Morph*x*Time | 1618.0  827.4  198.6 | ***  ***  *** |
| F_v_/F_m_ ratio  Morph  Time  Morph*x*Time | 0.5  14.2  0.1 | ns  ***  ns |
| F_0_ value  Morph  Time  Morph*x*Time | 15.5  581.6  13.8 | ***  ***  *** |
| Φ_PSII_  Morph  Time  Morph*x*Time | 17.3  384.6  6.8 | ***  ***  *** |
| NPQ  Morph  Time  Morph*x*Time | 300.1  423.3  3.9 | ***  ***  * |
| Chl_TOT_  Morph  Time  Morph*x*Time | 6.1  182.6  101.1 | *  ***  *** |
| β-carotene  Morph  Time  Morph*x*Time | 54.0  6.5  126.5 | ***  *  *** |
| VAZ/Chl_TOT_  Morph  Time  Morph*x*Time | 28.2  20.9  1.1 | ***  ***  ns |
| Hydrogen peroxide  Morph  Time  Morph*x*Time | 09.8  14334.0  1015.0 | ***  ***  *** |
| Superoxide anion  Morph  Time  Morph*x*Time | 386.3  189.6  386.1 | ***  ***  *** |
