# Supplementary figures and images for: Multiple Consequences Induced by Epidermally-Located Anthocyanins in Young, Mature and Senescent Leaves of Prunus
Source: Front Plant Sci. 2018 Jul 2;9:917. doi: 10.3389/fpls.2018.00917 (PMC6036500; doi:10.3389/fpls.2018.00917)

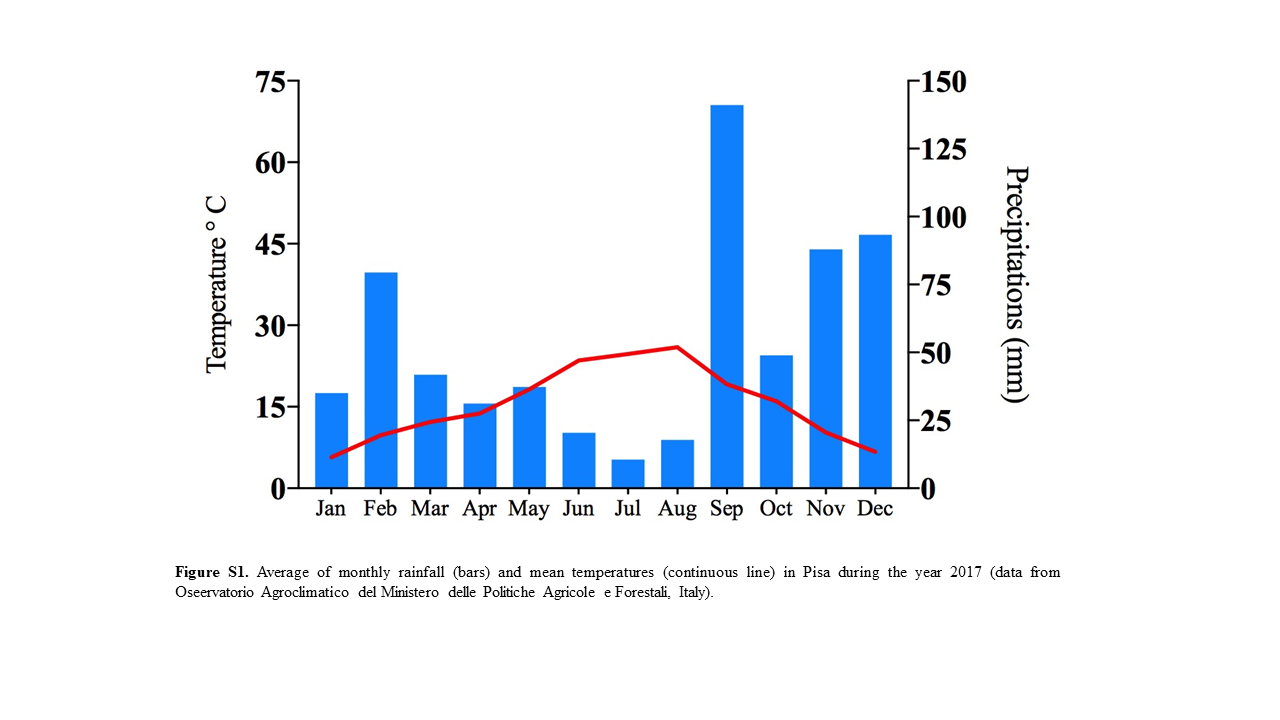

Supplement: Supplementary file 2 [file Image_1.TIF]

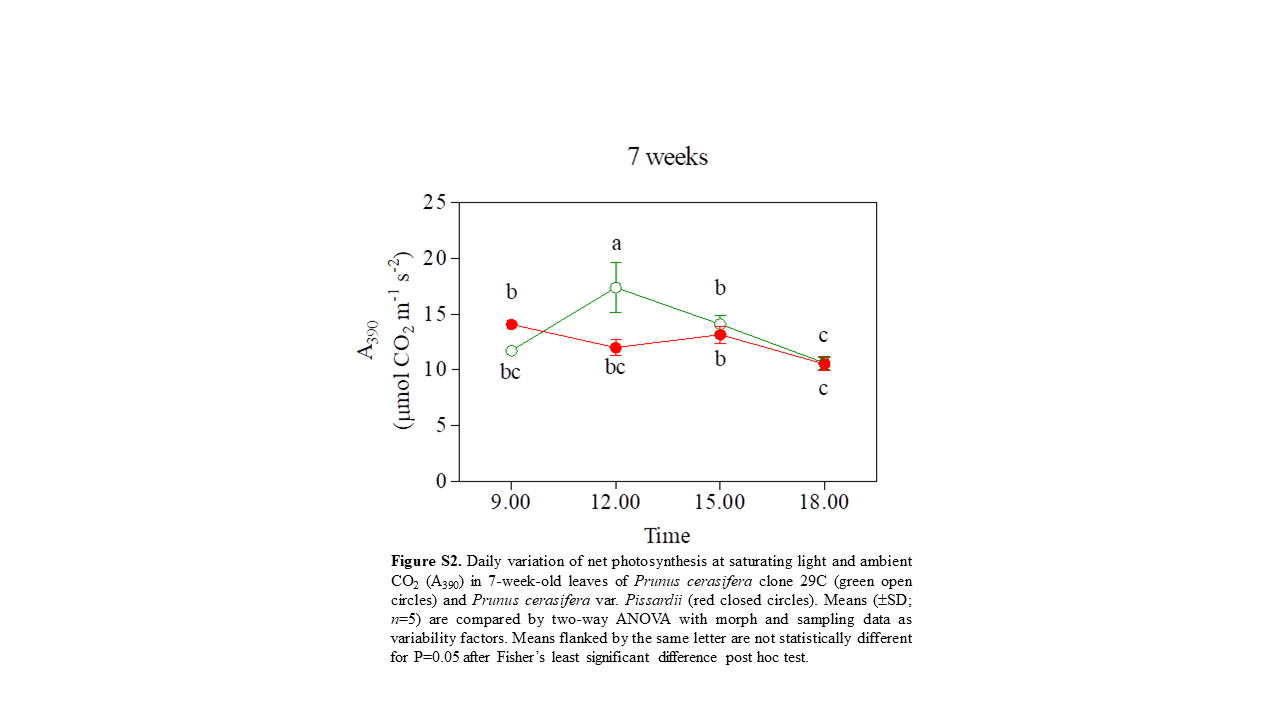

Supplement: Supplementary file 3 [file Image_2.TIF]

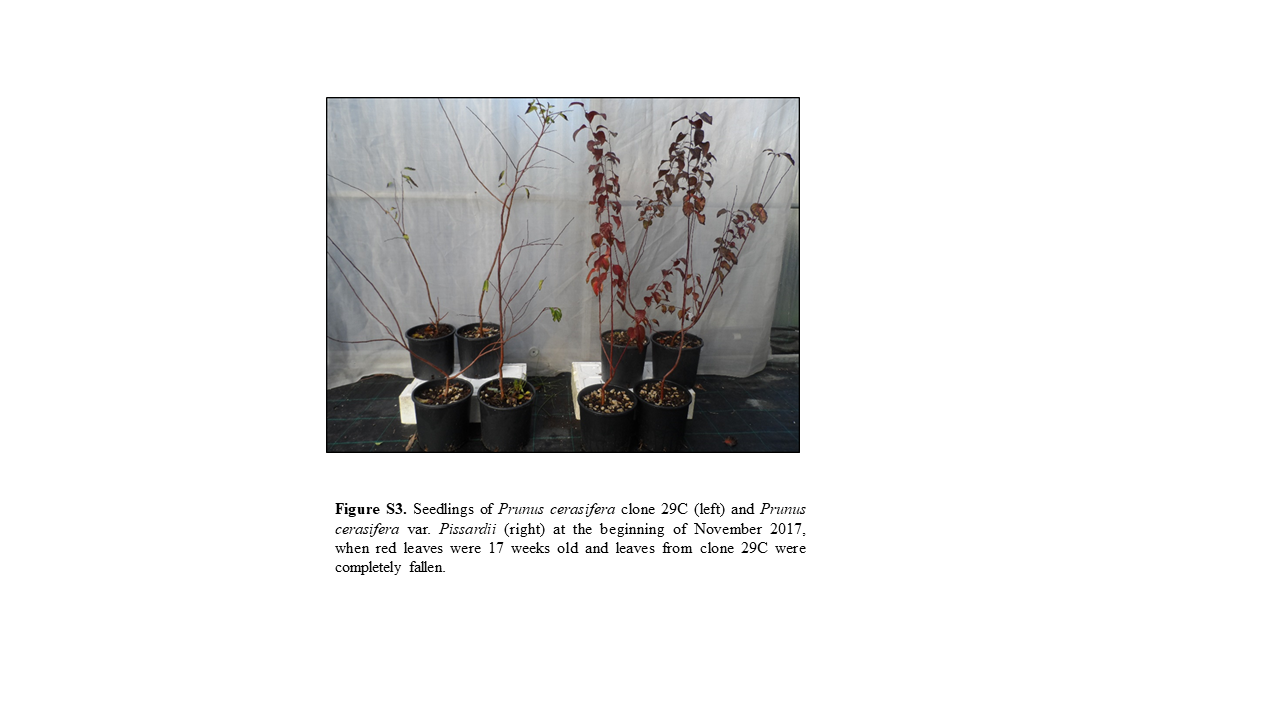

Supplement: Supplementary file 4 [file Image_3.TIF]
